# Supplementary material for: Pannexin-1 channel inhibition alleviates opioid withdrawal in rodents by modulating locus coeruleus to spinal cord circuitry
Source: Nat Commun. 2024 Jul 24;15:6264. doi: 10.1038/s41467-024-50657-7 (PMC11269731; doi:10.1038/s41467-024-50657-7)
Supplement: Supplementary file 1 — Supplementary Information [file 41467_2024_50657_MOESM1_ESM.pdf]

Supplementary Information:

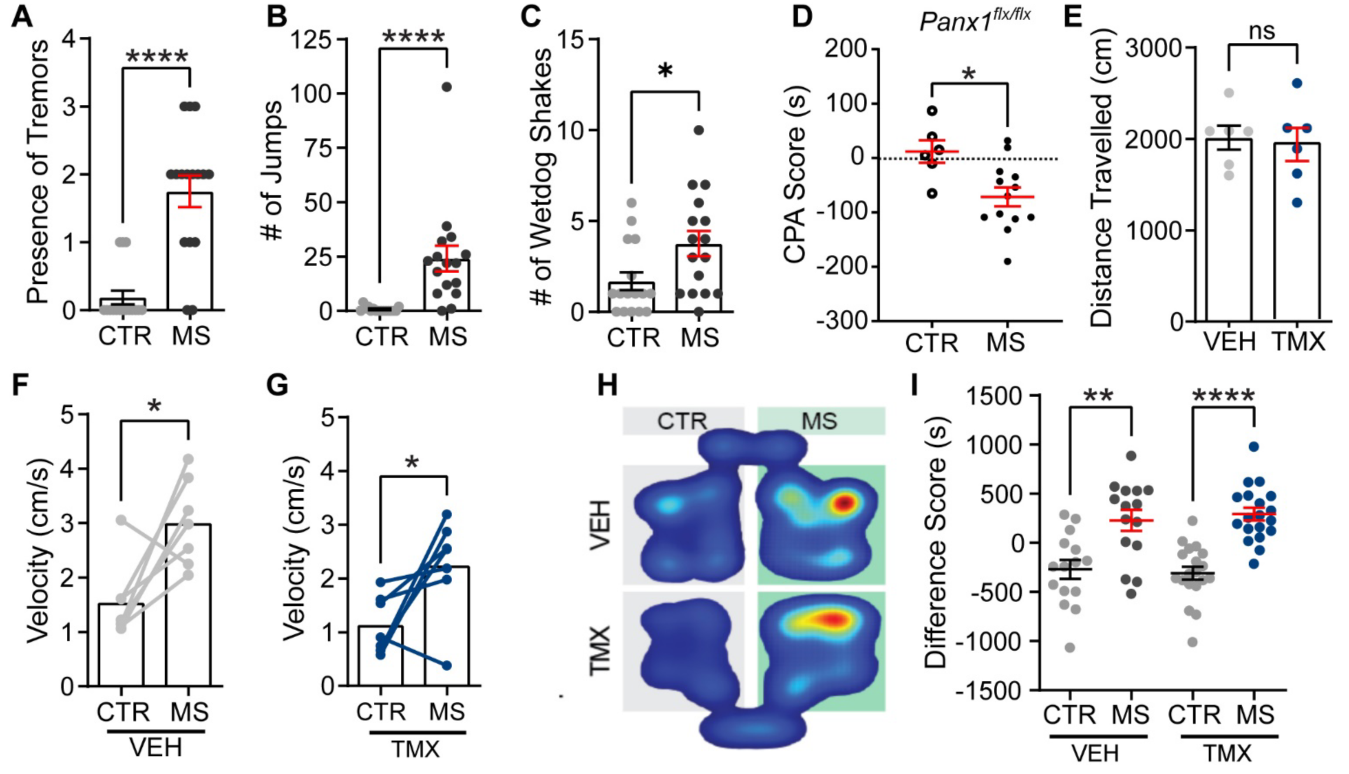

Supplementary Figure 1. Tamoxifen treatment alone has no effect on locomotion or morphine-induced conditioned place preference.

(**A-C**) Quantification of somatic withdrawal behaviours in saline (CTR, N=16 mice) and morphine (MS, N=16 mice) treated mice during CPA following injection of naloxone. (**A**) Presence of tremors (Mann-Whitney two-sided test, $p<0.0001$). (**B**) Number of instances of jumping (Mann-Whitney two-sided test, $p<0.0001$). (**C**) Number of instances of wet-dog shakes (Mann-Whitney two-sided test, $p=0.019$). (**D**) Naloxone-induced CPA in TMX treated Panx1$^{flx/flx}$ mice at day 1 post-conditioning (unpaired two-sided t-test, $p=0.0109$) (CTR: N=6, MS: N=13 mice). (**E**) Locomotor ability measured by total distance traveled (cm) of drug naïve vehicle (VEH) and tamoxifen (TMX) *Cx3cr1*-Cre^{ERT2}::*Panx1*$^{flx/flx}$ mice in open-field area (unpaired two-sided t-test, $p=0.7492$, VEH: N=6, TMX: N=6 mice). (**F-G**) Locomotor velocity (cm/s) in drug naïve state (CTR) and after acute morphine (MS) injection in VEH (Wilcoxon matched-pairs signed rank two-sided test, $p=0.0313$, N=7 mice) and TMX (paired two-sided t-test, $p=0.0429$, N=7 mice) treated *Cx3cr1*-Cre^{ERT2}::*Panx1*$^{flx/flx}$ mice. (**H**) Representative heat maps of total time spent by VEH and TMX *Cx3cr1*-Cre^{ERT2}::*Panx1*$^{flx/flx}$ mice during MS

conditioned place preference (CPP) test. (**I**) Quantification of morphine treatment CPP in VEH and TMX treated *Cx3cr1*-Cre^{ERT2}::*Panx1*$^{flx/flx}$ mice (two-way RM ANOVA, VEH or TMX treatment: p= 0.8535, morphine treatment, p<0.0001, VEH: N=15, TMX: N=19 mice). All graphed data are presented as mean values ±SEM. *p<0.05, **p<0.01, ***p<0.001, ****p<0.0001. Source data are provided as a Source Data file.

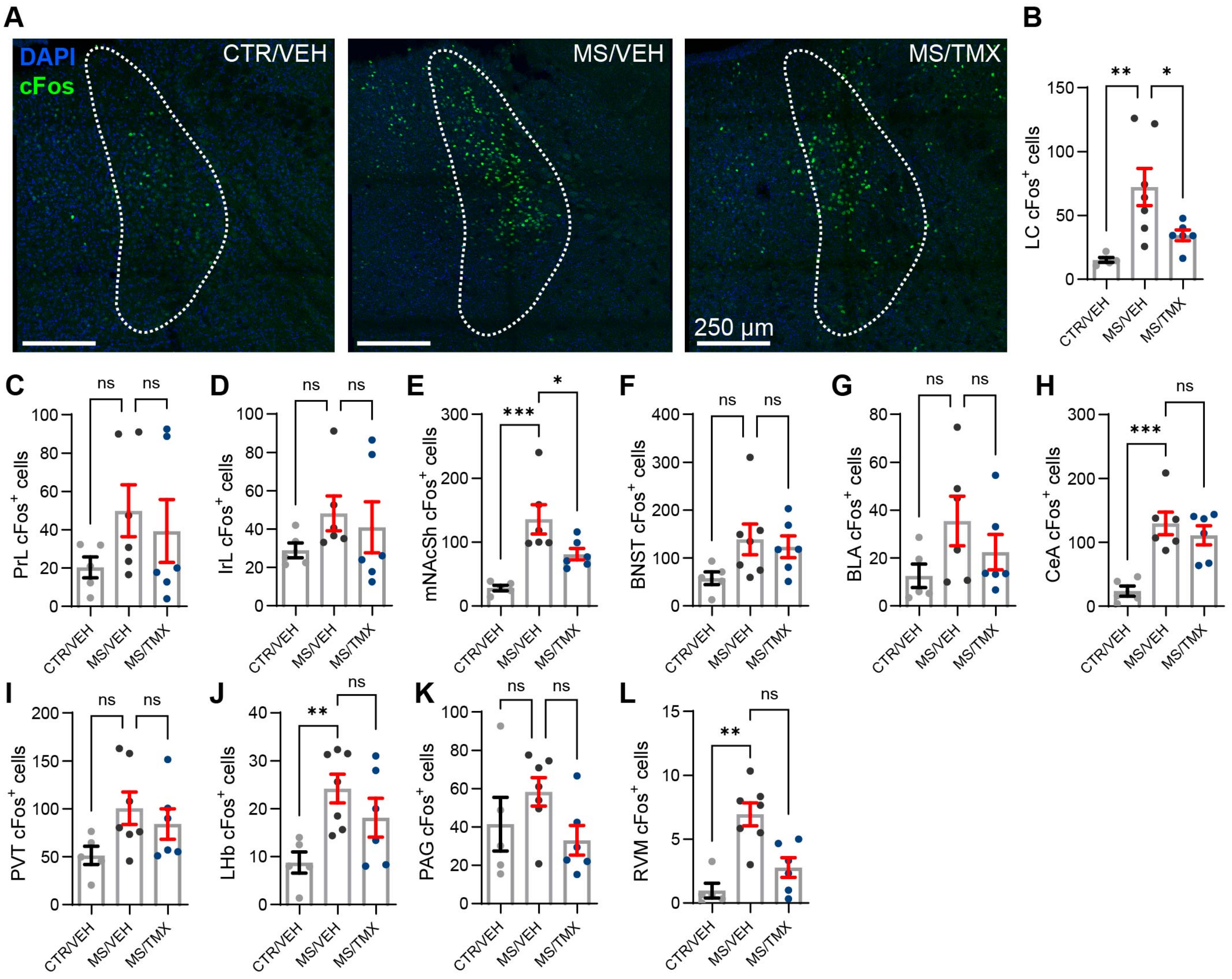

Supplementary Figure 2. Whole brain analysis reveals the locus coeruleus as a key region of difference between wild-type and microglial-Panx1 deficient mice during opioid withdrawal.

(**A**) Representative LC images of cFos immunoreactivity in coronal brain sections from naloxone challenged saline control (CTR) vehicle treated (CTR/VEH) and morphine (MS) withdrawn vehicle (MS/VEH) or tamoxifen (MS/TMX) treated *Cx3cr1*-Cre^{ERT2}::*Panx1*$^{flx/flx}$ mice. (**B to L**) Quantification of cFos+ cells during opioid withdrawal in CTR/VEH, MS/VEH, and MS/TMX *Cx3cr1*-Cre^{ERT2}::*Panx1*$^{flx/flx}$ mice. (**B**) Locus coeruleus (LC) (one-way ANOVA, p=0.0042, CTR/VEH, MS/VEH, MS/TMX: N=5, 7, 6). (**C**) Prelimbic cortex (PrL) (one-way ANOVA, p=0.3338, CTR/VEH, MS/VEH, MS/TMX: N=5, 6, 6 mice). (**D**) Infralimbic cortex (IrL) (Kruskal-Wallis test, p=0.1935, CTR/VEH, MS/VEH, MS/TMX: N=5, 6, 6 mice). (**E**) Medial shell of the nucleus accumbens (mNAcSh) (one-way ANOVA, p=0.0009, CTR/VEH, MS/VEH,

MS/TMX: N=5, 6, 6 mice). (**F**) Bed nucleus of the stria terminalis (BNST) (one-way ANOVA, p=0.1176, CTR/VEH, MS/VEH, MS/TMX: N=5, 7, 6 mice). (**G**) Basolateral amygdala (BLA) (one-way ANOVA, p=0.1850, CTR/VEH, MS/VEH, MS/TMX: N=5, 6, 6 mice). (**H**) Central amygdala (CeA) (one-way ANOVA, p=0.0005, CTR/VEH, MS/VEH, MS/TMX: N=5, 6, 6 mice). (**I**) Paraventricular thalamus (PVT) (one-way ANOVA, p=0.1143, CTR/VEH, MS/VEH, MS/TMX: N=5, 7, 6 mice). (**J**) Lateral habenula (LHb) (one-way ANOVA, p=0.0167, CTR/VEH, MS/VEH, MS/TMX: N=5, 7, 6 mice). (**K**) Periaqueductal gray (PAG) (one-way ANOVA, p=0.1683, CTR/VEH, MS/VEH, MS/TMX: N=5, 7, 6 mice). (**L**) Rostral ventromedial medulla (RVM) (Kruskal-Wallis test, p=0.0006, N=5, 7, 6 mice). All graphed data are presented as mean values ±SEM. *p<0.05, **p<0.01, ***p<0.001, ****p<0.0001. Source data are provided as a Source Data file.

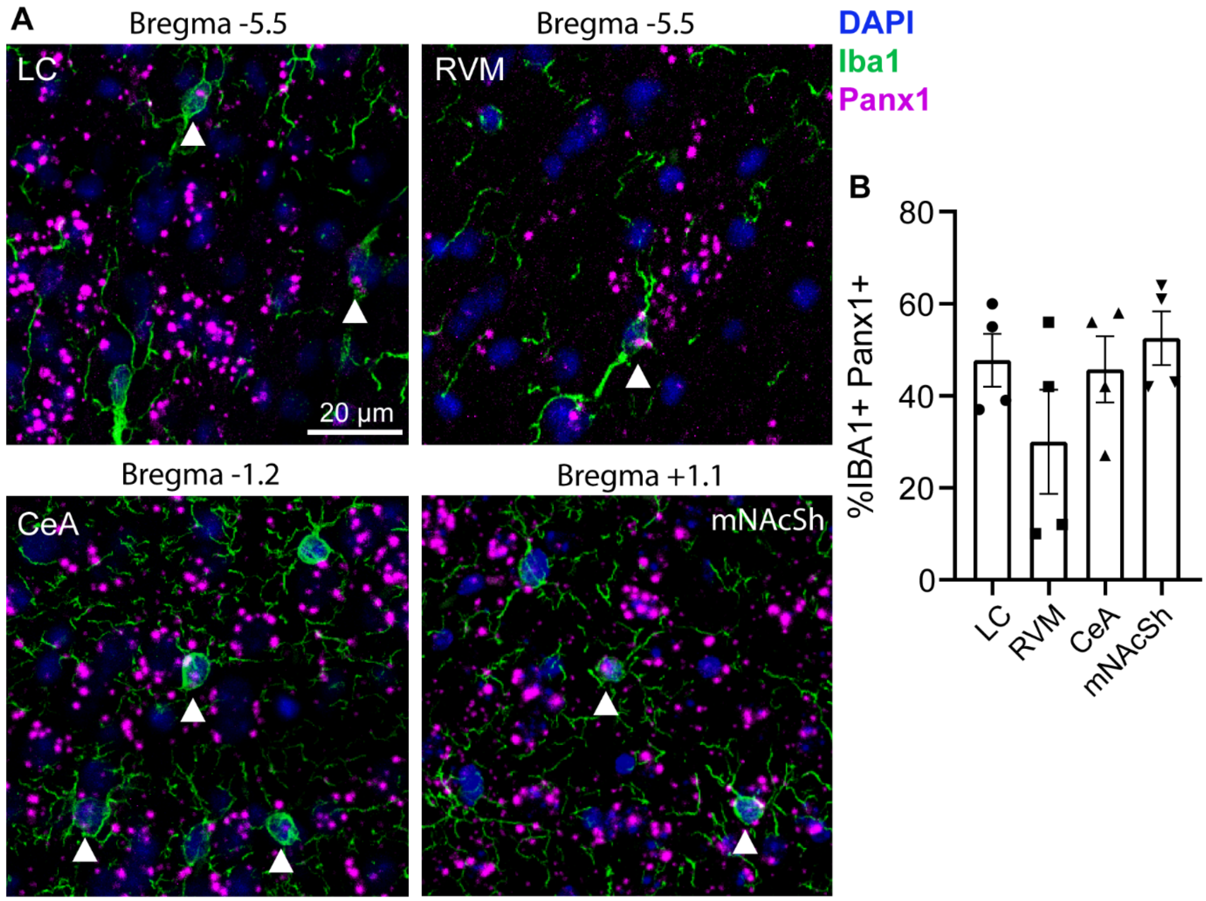

Supplementary Figure 3. Whole brain analysis reveals *Panx1* is expressed in locus coeruleus microglia.

(A) Representative images of locus coeruleus (LC), rostral ventromedial medulla (RVM), central amygdala (CeA), and medial shell of the nucleus accumbens (mNAcSh) of C57Bl/6 mice labelled for Panx1 mRNA using RNAscope 2.5 HD Assay RED, and IBA1 using subsequent immunohistochemistry. B) Quantification of % of IBA1+ cells that contain mRNA for Panx1 (LC: N= 27 slices/4 mice (16±1 microglia per slice), RVM: N=16 slices/4 mice (10±1 microglia per slice), CeA: N=24 slices/4 mice (52±3 microglia per slice), mNAcSh: N=22 slices/4 mice (48±4 microglia per slice)). All graphed data are presented as mean values ±SEM. Source data are provided as a Source Data file.

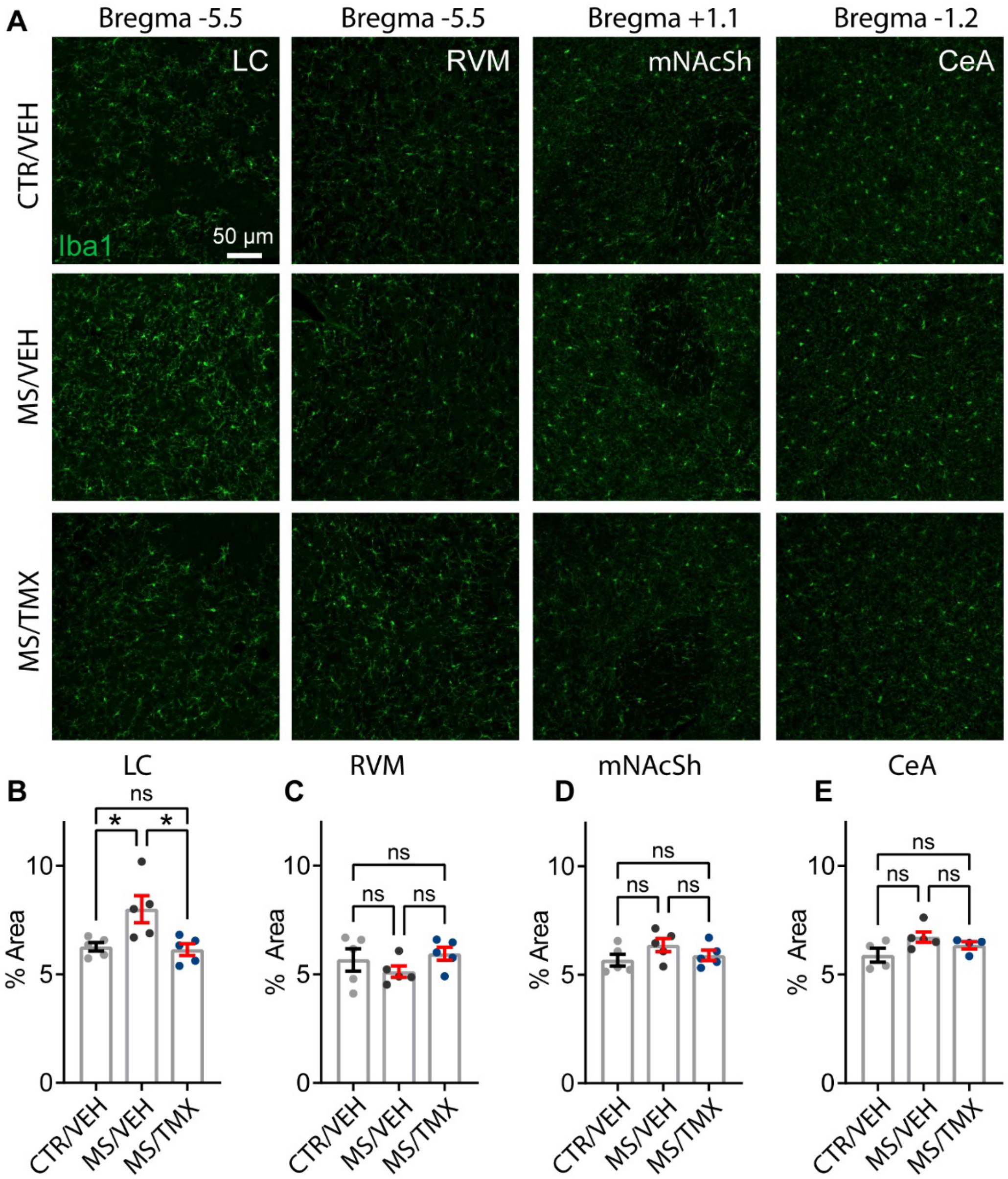

Supplementary Figure 4. Increase in Iba1 staining of the locus coeruleus during opioid withdrawal is dependent on microglial Panx1.

(**A**) Representative images and quantification of Iba1 immunostaining in saline (CTR) and morphine (MS) treated, vehicle treated (CTR/VEH and MS/VEH) *Cx3cr1*-Cre^{ERT2}::*Panx1*$^{flx/flx}$ mice and morphine treated, tamoxifen treated (MS/TMX) *Cx3cr1*-Cre^{ERT2}::*Panx1*$^{flx/flx}$ mice. (**B**) Quantification of Iba1 % area in locus coeruleus (LC) (one-way ANOVA, p=0.0124, N=5 mice in each group). (**C**) Quantification of Iba1 % area in rostral ventromedial medulla (RVM) (one-

way ANOVA, p=0.3198, N=5 mice in each group). (**D**) Quantification of Iba1 % area in the medial shell of the nucleus accumbens (mNAcSh) (one-way ANOVA, p=0.2291, N=5 mice in each group). (E) Quantification of Iba1 % area in the central amygdala (CeA) (one-way ANOVA, p=0.3215, CTR/VEH: N=4, MS/VEH: N=5, MS/TMX: N=5 mice). All graphed data are presented as mean values ±SEM. *p<0.05. Source data are provided as a Source Data file.

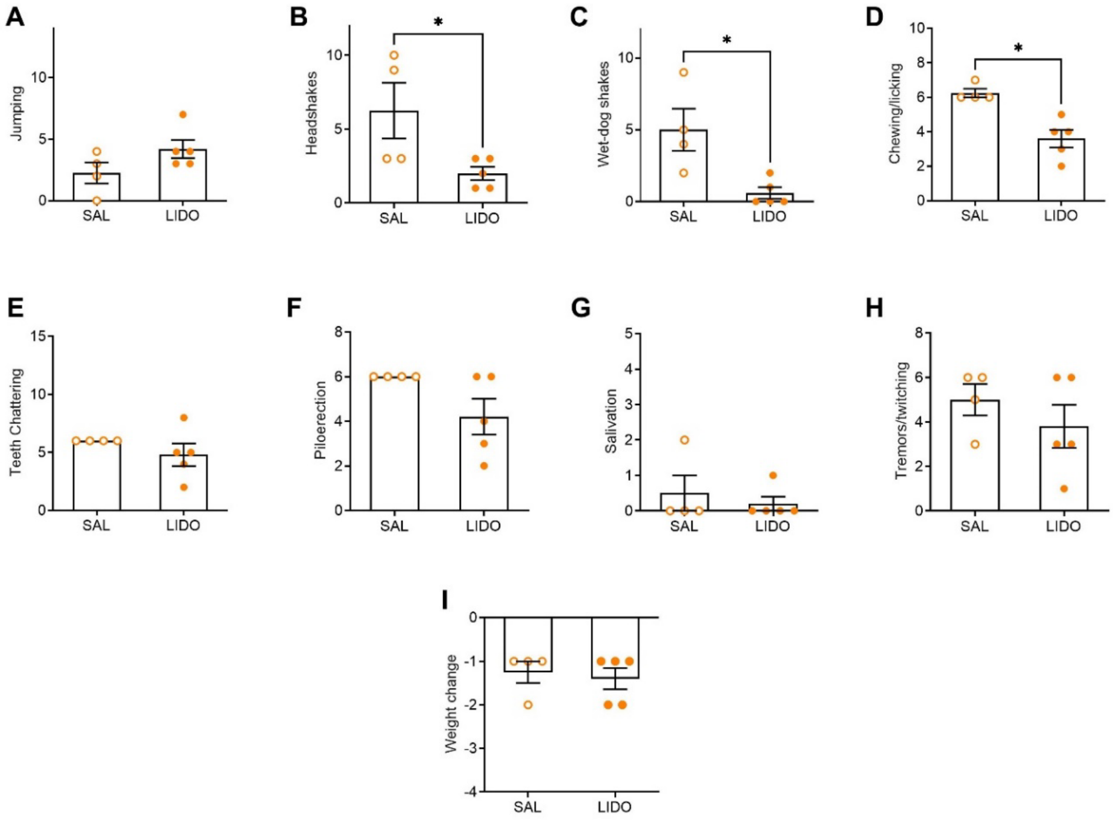

Supplementary Figure 5: Individual withdrawal behaviours in saline and lidocaine intracerebral LC treated mice.

Individual behaviour counts over a 30-minute period used to calculate cumulative withdrawal scores in saline (SAL) and Lidocaine (LIDO, 1%) (intra-LC cannula administration) treated morphine-dependent mice undergoing naloxone-precipitated withdrawal. (**A**) Total counts of jumping (unpaired two-sided t-test, p=0.125). (**B**) Total counts of headshakes (unpaired two-sided t-test, p=0.0440). (**C**) Total counts of wet-dog shakes (Mann-Whitney two-sided test, p=0.0238). (**D**) Total counts of chewing and licking (Mann-Whitney two-sided test, p=0.0159). (**E**) Total counts of teeth chattering (Mann-Whitney two-sided test, p=0.111). (**F**) Presence of piloerection in each five-minute bin (Mann-Whitney two-sided test, p=0.167). (**G**) Presence of salivation in each five-minute bin (Mann-Whitney two-sided test, p=0.722). (**H**) Presence of tremors or twitching in each five-minute bin (unpaired two-sided t-test, p=0.374). (**I**) Total weight change (Mann-Whitney two-sided test, p>0.999). For all behaviours, SAL N=4, LIDO N=5. All graphed data are presented as mean values ±SEM. *p<0.05. Source data are provided as a Source Data file.

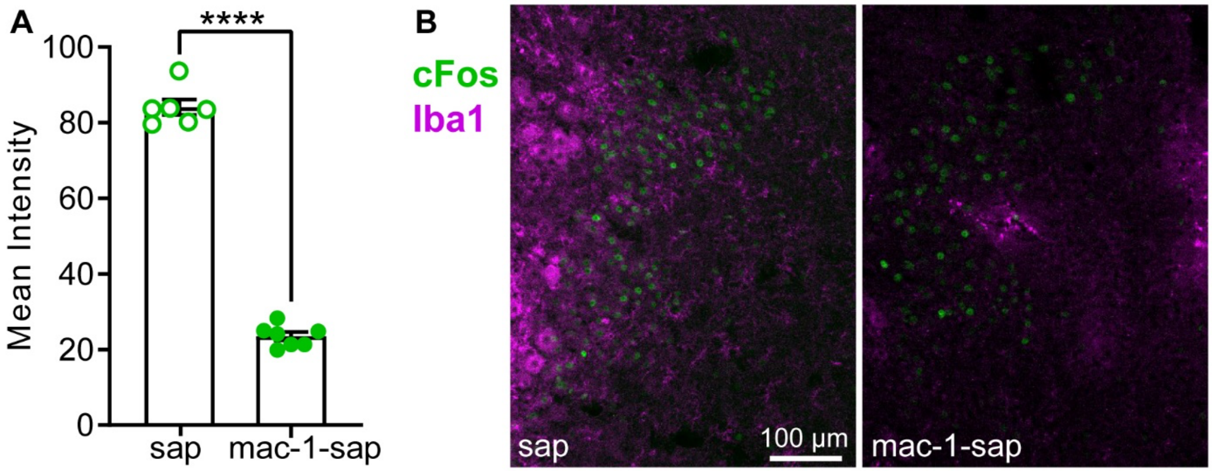

Supplementary Figure 6. Intracerebral delivery of Mac-1-saporin successfully depletes LC microglia.

(A) Comparison of mean intensity of Iba1 immunostaining in the LC between mice treated for three consecutive days with saporin (sap) or Mac-1-saporin (mac-1-sap, 15 µg) administered through a bilateral cannula (unpaired two-sided t-test, $p<0.0001$, Sap: N=6, mac-1-sap: N=7 mice). (B) Representative images of Iba1 immunostaining from in the LC after treatment with sap (left) or mac-1-sap (right) (Sap: N=6, mac-1-sap: N=7 mice). All graphed data are presented as mean values ±SEM. ****$p<0.0001$. Source data are provided as a Source Data file.

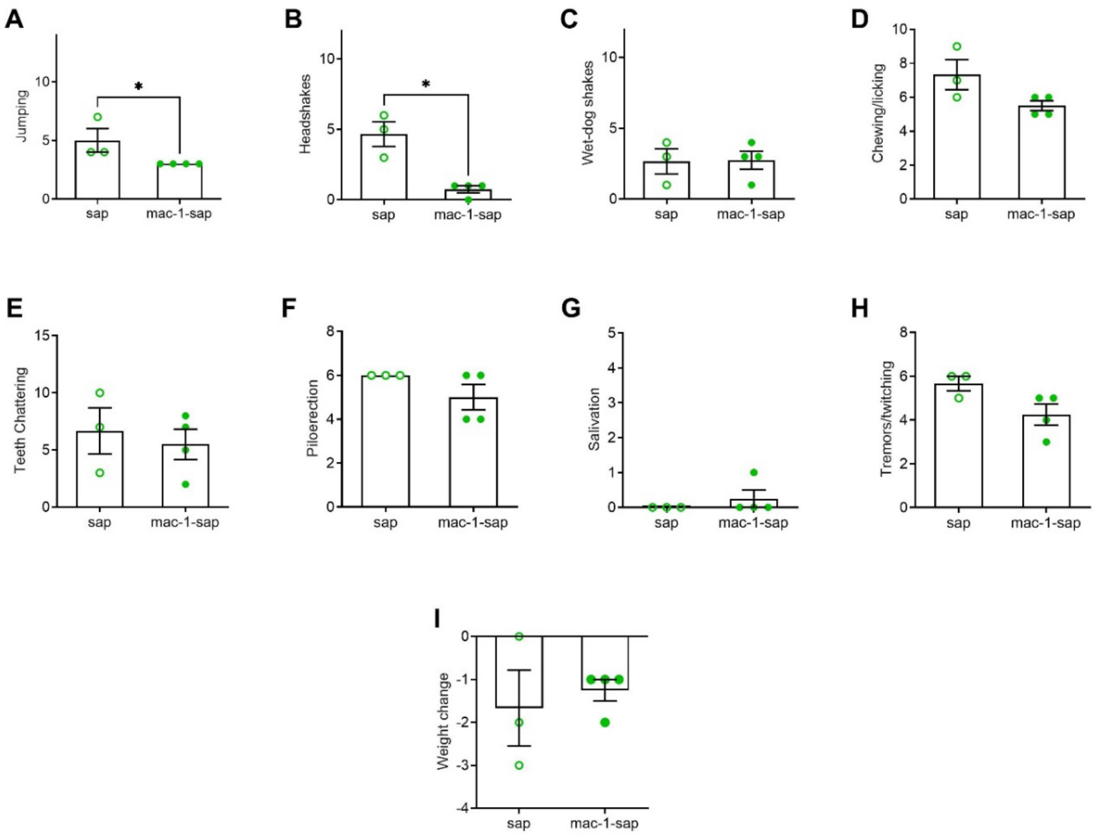

Supplementary Figure 7: Individual withdrawal behaviours in saporin and mac-1-saporin intracerebral LC treated mice.

Individual behaviour counts over a 30-minute period used to calculate cumulative withdrawal scores in saporin (sap) and mac-1-saporin (mac-1-sap, 15 µg) (intra-LC cannula administration) treated morphine-dependent mice undergoing naloxone-precipitated withdrawal. (**A**) Total counts of jumping (Mann-Whitney two-sided test, p=0.0286). (**B**) Total counts of headshakes (Mann-Whitney two-sided test, p=0.0286). (**C**) Total counts of wet-dog shakes (unpaired two-sided t-test, p=0.940). (**D**) Total counts of chewing and licking (Mann-Whitney two-sided test, p=0.171). (**E**) Total counts of teeth chattering (unpaired two-sided t-test, p=0.635). (**F**) Presence of piloerection in each five-minute bin (Mann-Whitney two-sided test, p=0.429). (**G**) Presence of salivation in each five-minute bin (Mann-Whitney two-sided test, p>0.999). (**H**) Presence of tremors or twitching in each five-minute bin (unpaired two-sided t-test, p=0.0749). (**I**) Total weight change (Mann-Whitney two-sided test, p=0.629). For all behaviours, sap N=3, mac-1-sap N=4. All graphed data are presented as mean values ±SEM. *p<0.05. Source data are provided as a Source Data file.

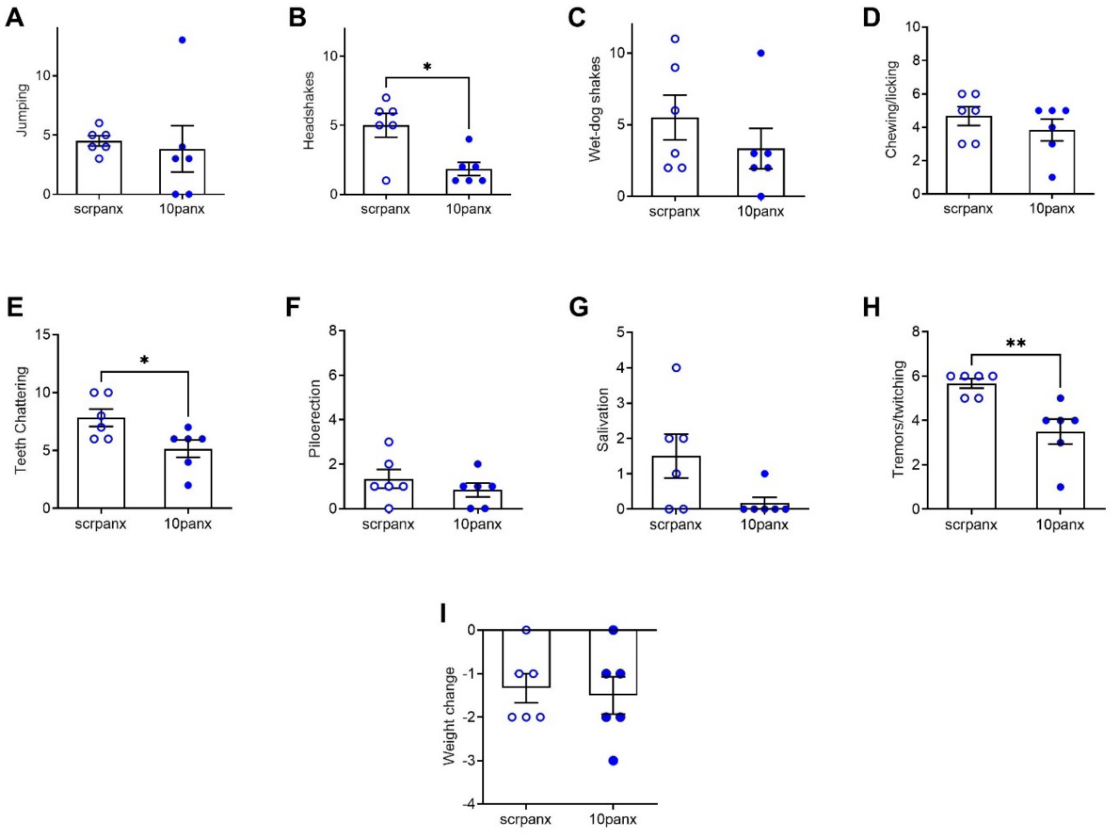

Supplementary Figure 8: Individual withdrawal behaviours in ^{scr}Panx and ^{10}Panx intracerebral LC treated mice.

Individual behaviour counts over a 30-minute period used to calculate cumulative withdrawal scores in scrambled ^{10}Panx (^{scr}Panx) and ^{10}Panx (^{10}Panx (10 μg)) (intra-LC cannula administration) treated morphine-dependent mice undergoing naloxone-precipitated withdrawal. (**A**) Total counts of jumping (Mann-Whitney two-sided test, p=0.165). (**B**) Total counts of headshakes (Mann-Whitney two-sided test, p=0.0281). (**C**) Total counts of wet-dog shakes (Mann-Whitney two-sided test, p=0.353). (**D**) Total counts of chewing and licking (Mann-Whitney two-sided test, p=0.355). (**E**) Total counts of teeth chattering (unpaired two-sided t-test, p=0.0305). (**F**) Presence of piloerection in each five-minute bin (unpaired two-sided t-test, p=0.361). (**G**) Presence of salivation in each five-minute bin (Mann-Whitney two-sided test, p=0.106). (**H**) Presence of tremors or twitching in each five-minute bin (Mann-Whitney two-sided test, p=0.0065). (**I**) Total weight change (unpaired two-sided t-test, p=0.765). For all behaviours, ^{scr}Panx N=6, ^{10}Panx N=6. All graphed data are presented as mean values ±SEM. *p<0.05. Source data are provided as a Source Data file.

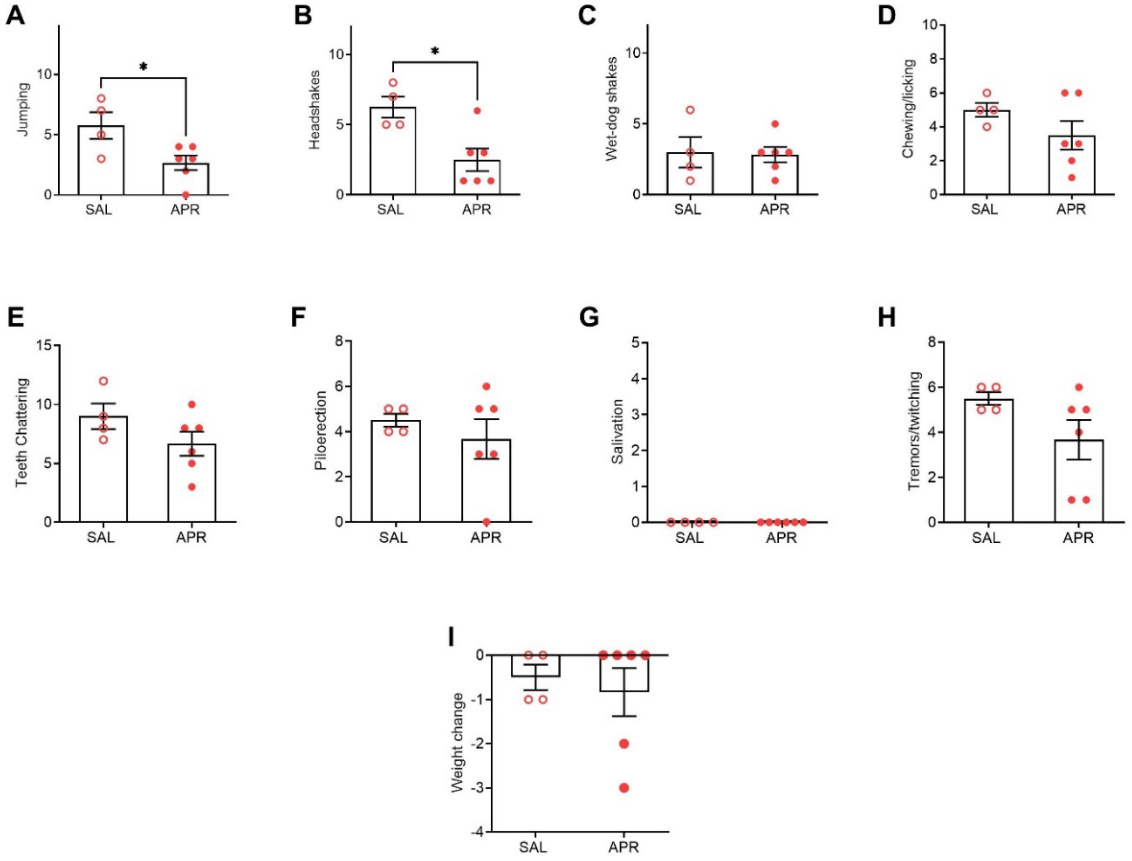

Supplementary Figure 9: Individual withdrawal behaviours in saline and apyrase intracerebral LC treated mice.

Individual behaviour counts over a 30-minute period used to calculate cumulative withdrawal scores in saline (SAL) and apyrase (APR, 10 units) (intra-LC cannula administration) treated morphine-dependent mice undergoing naloxone-precipitated withdrawal. (**A**) Total counts of jumping (unpaired two-sided t-test, p=0.0295). (**B**) Total counts of headshakes (unpaired two-sided t-test, p=0.0125). (**C**) Total counts of wet-dog shakes (unpaired two-sided t-test, p=0.882). (**D**) Total counts of chewing and licking (unpaired two-sided t-test, p=0.212). (**E**) Total counts of teeth chattering (unpaired two-sided t-test, p=0.167). (**F**) Presence of piloerection in each five-minute bin (Mann-Whitney two-sided test, p=0.705). (**G**) Presence of salivation in each five-minute bin (Mann-Whitney two-sided test, p>0.999). (**H**) Presence of tremors or twitching in each five-minute bin (Mann-Whitney two-sided test, p=0.171). (**I**) Total weight change (Mann-Whitney two-sided test, p>0.999). For all behaviours, SAL N=4, APR N=6. All graphed data are presented as mean values ±SEM. *p<0.05. Source data are provided as a Source Data file.

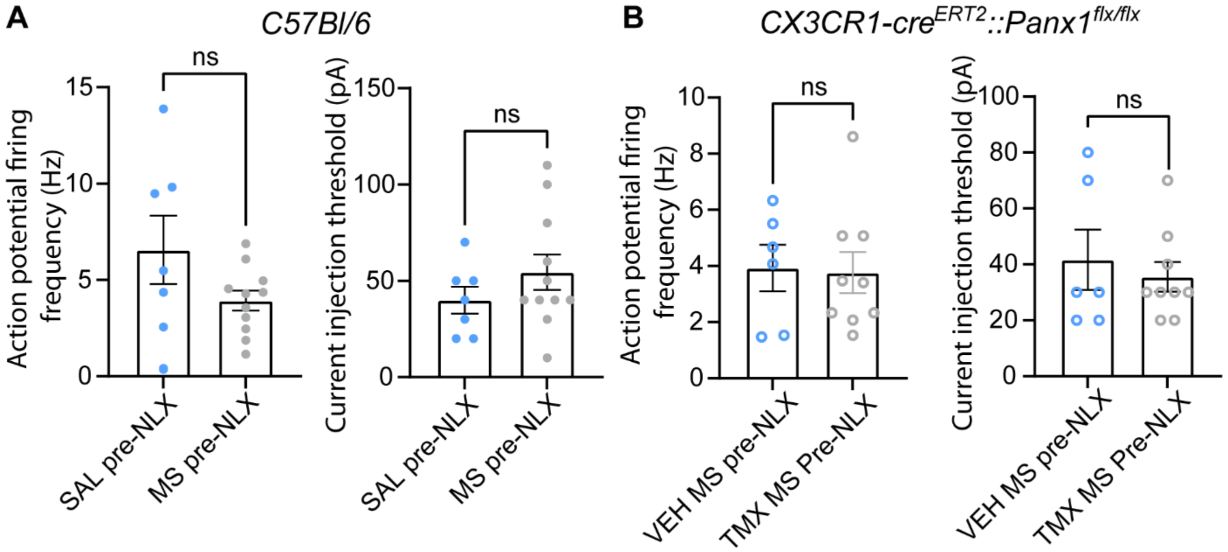

Supplementary Figure 10: Basal LC neuronal activity is unaffected by morphine treatment.

(**A**) Left: Action potential firing frequency of LC neurons in response to a 3000 ms, 100 pA ramp depolarization in morphine (MS) and saline (SAL) treated mice (unpaired two-sided t-test, p=0.1026, MS N=11 neurons/9 mice, SAL N=8 neurons/7 mice). Right: Current injection threshold for action potential firing in response to square depolarization for 1000 ms in morphine (MS) and saline (SAL) treated mice (unpaired two-sided t-test, p=0.2737, MS N=11 neurons/8 mice, SAL N=7 neurons/6 mice). (**B**) Action potential frequency in *Cx3cr1*-Cre^{ERT2}::*Panx1*$^{flx/flx}$ mice (unpaired two-sided t-test, p=0.8861, VEH MS N=6 neurons/5 mice, TMX MS N=9 neurons/7 mice) and current injection threshold (unpaired two-sided t-test, p=0.5827, VEH MS N=6 neurons/5 mice, TMX MS N=9 neurons/7 mice) between vehicle (VEH) and tamoxifen (TMX) morphine treated mice. All graphed data are presented as mean values ±SEM. Source data are provided as a Source Data file.

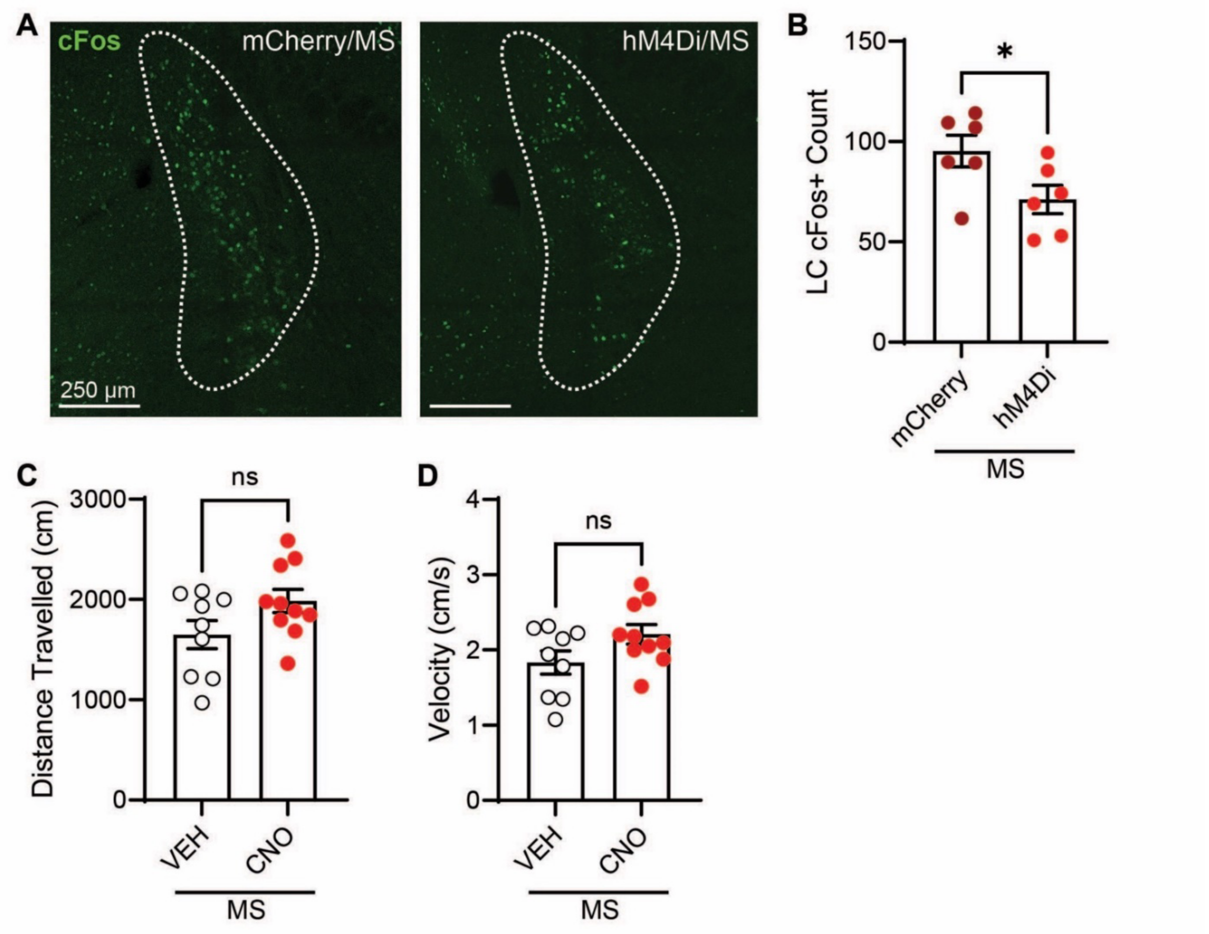

Supplementary Figure 11: Chemogenetic silencing of LC^{spinal} neurons reduces cFos response during morphine withdrawal without affecting locomotor activity.

(**A**) Representative LC images of cFos immunoreactivity from morphine treated mice expressing mCherry or inhibitory DREADD (hM4Di) after naloxone induced withdrawal. Mice were treated with CNO (1 mg/kg; i.p.) prior to naloxone challenge. (mCherry: N=6, hM4Di: N=6 mice) (**B**) Quantificaiton of LC cFos^{+} cell counts after naloxone administration (unpaired two-sided t-test, p=0.0470, mCherry: N=6, hM4Di: N=6 mice). (**C**) Locomotor activity measured by total distance traveled (cm) and (**D**) velocity (cm/s) in the CPA test. Morphine treated hM4Di expressing mice were administered vehicle or CNO prior to naloxone challenge. (Distance Travelled: unpaired two-sided t-test, p=0.0794; Velocity: unpaired two-sided t-test, p=0.0785; VEH: N=9, CNO: N=10 mice). All graphed data are presented as mean values ±SEM. *p<0.05. Source data are provided as a Source Data file.

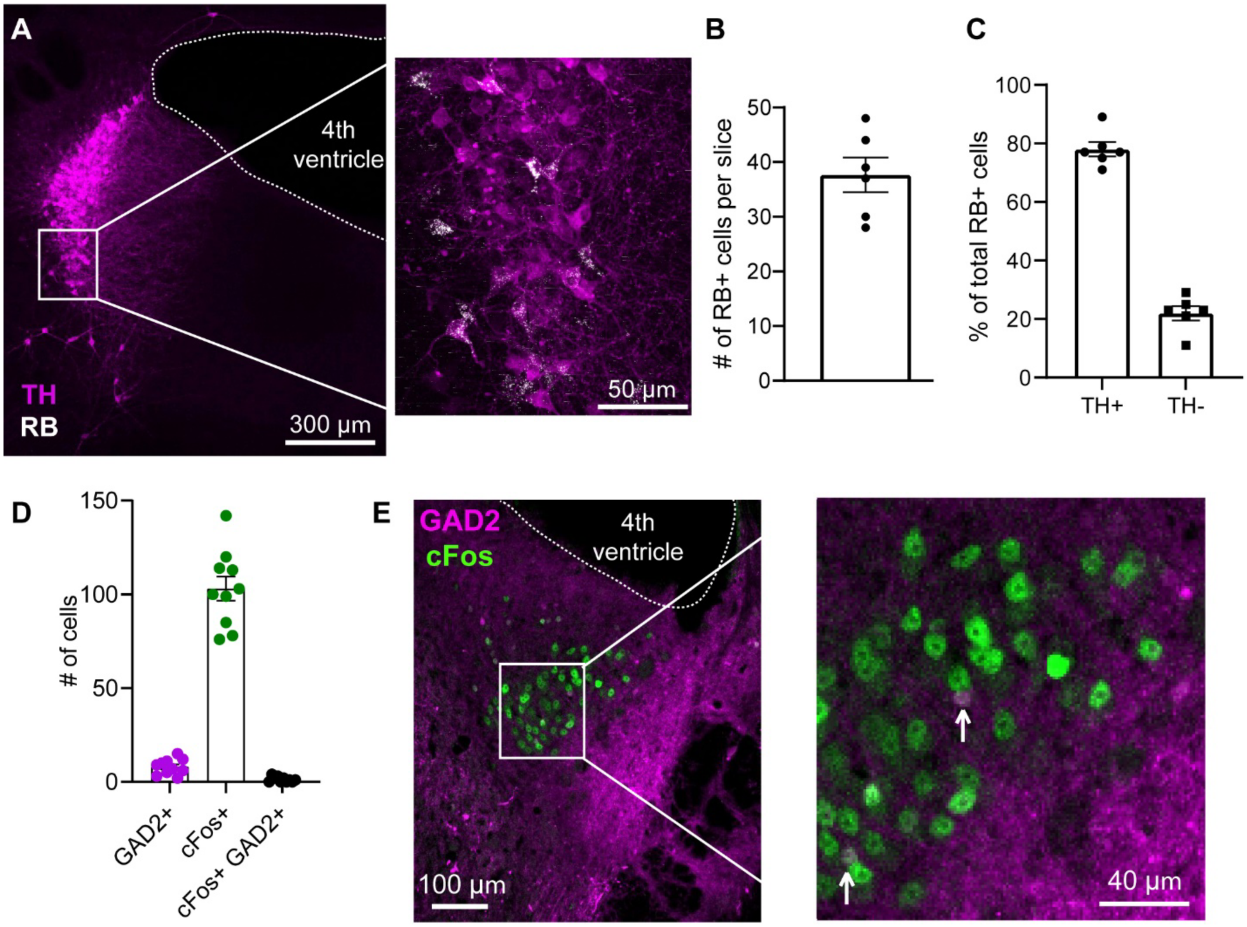

Supplementary Figure 12: The majority of LC neurons projecting to the spinal cord are monoaminergic.

(**A**) Representative images of retrobead (RB, white) labelling and tyrosine-hydroxylase (TH, magenta) immunoreactivity in the LC. (N=6 slices/6 mice). (**B**) Quantification of total number of RB+ neurons in each LC slice (unilateral) (N=6 slices/6 mice). (**C**) TH and RB colocalization within the LC (N=6 slices/6 mice). (**D**) Quantification of cFos and GAD2-ai9 colocalization (N=10 slices/5 mice). (**E**) Representative image and magnification of cFos and GAD2-ai9 colocalization. (N=10 slices/5 mice). All graphed data are presented as mean values ±SEM. Source data are provided as a Source Data file.

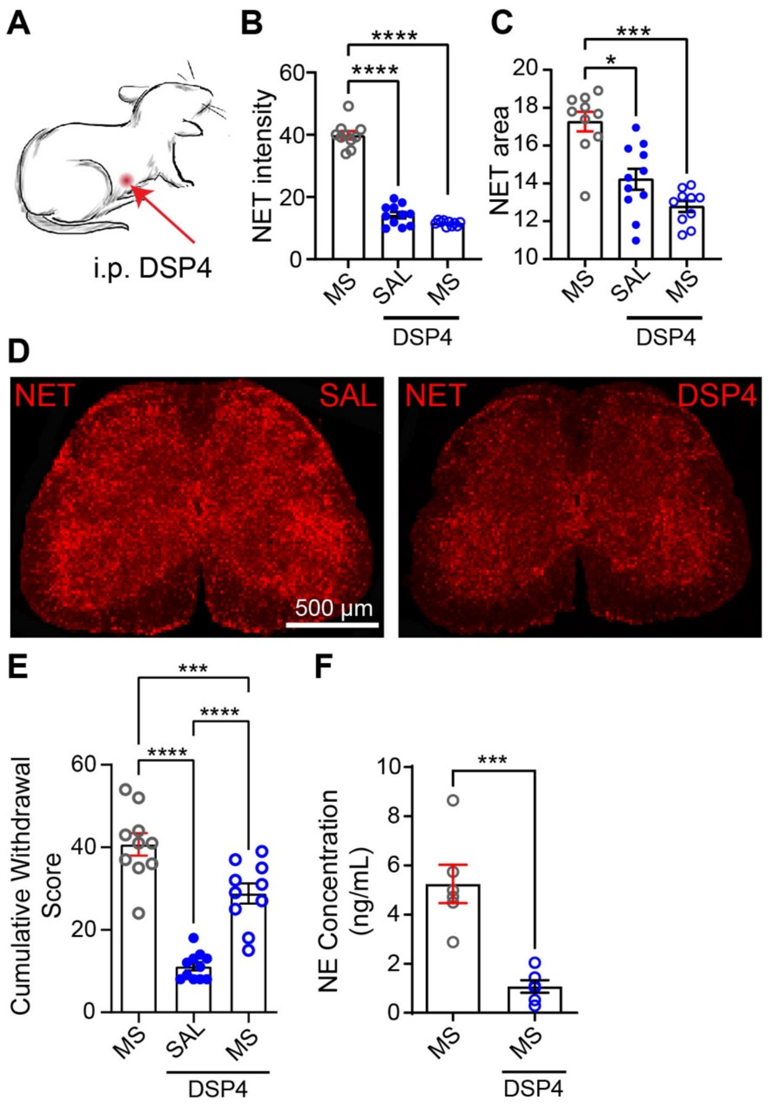

Supplementary Figure 13: Systemic DSP4 administration reduces spinally-projecting monoaminergic neurons

(**A**) Schematic depicting N-(2-chloroethyl)-N-ethyl-2-bromobenzylamine hydrochloride (DSP4, 50 mg/kg, i.p.) administration. (**B and C**) Quantification of NET immunoreactivity in mice treated with i.p. DSP4 (SAL-MS, DSP4-MS: N=10, DSP4-SAL: N=11 mice) (**B**) NET intensity (one-way ANOVA, $p<0.0001$). (**C**) NET area (Kruskall-Wallis test, $p=0.0002$). (**D**) Representative immunostaining of norepinephrine transporter (NET) in DSP4 and saline (SAL) treated animals. (SAL-MS: N=10, DSP4-SAL: N=11 mice). (**E**) Cumulative withdrawal scores in mice treated with i.p. DSP4 (one-way ANOVA, $p<0.0001$, SAL-MS, DSP4-MS: N=10, DSP4-SAL: N=11 mice). (**F**) NE ELISA from DSP4 treated animals ($p=0.0005$, unpaired two-sided t

test, DSP4, Sal: N=6 mice). All graphed data are presented as mean values ±SEM. *p<0.05, **p<0.01, ***p<0.001, ****p<0.0001. Source data are provided as a Source Data file.

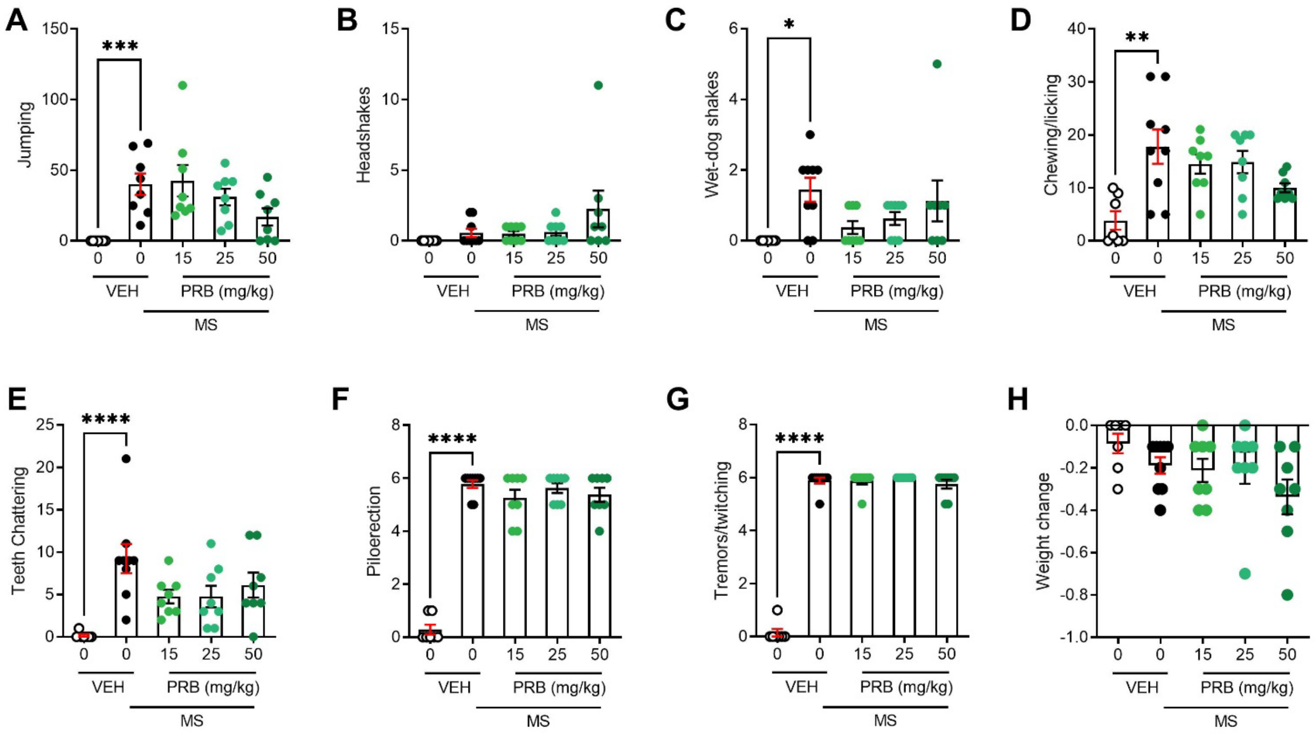

Supplementary Figure 14: Individual withdrawal behaviours in vehicle and probenecid treated mice.

Individual behaviour counts over a 30-minute period used to calculate cumulative withdrawal scores in vehicle (VEH) and Probenecid (i.p.) treated morphine-dependent mice undergoing naloxone-precipitated withdrawal. (**A**) Total counts of jumping (Kruskall-Wallis test, p=0.0007). (**B**) Total counts of headshakes (Kruskall-Wallis test, p=0.124). (**C**) Total counts of wet-dog shakes (one-way ANOVA, p=0.031). (**D**) Total counts of chewing and licking (Kruskall-Wallis test, p=0.0049). (**E**) Total counts of teeth chattering (one-way ANOVA, p=0.031). (**F**) Presence of piloerection in each five-minute bin (Kruskall-Wallis test, p=0.003). (**G**) Presence of tremors or twitching in each five-minute bin (one-way ANOVA, p<0.0001). (**H**) Total weight change (one-way ANOVA, p=0.117). For all behaviours, VEH-saline N=7, VEH-morphine (MS), PRB-MS, 15 mg/kg, 25 mg/kg, 50 mg/kg: N=8 mice. All graphed data are presented as mean values ±SEM. *p<0.05, **p<0.01, ***p<0.001, ****p<0.0001. Source data are provided as a Source Data file.

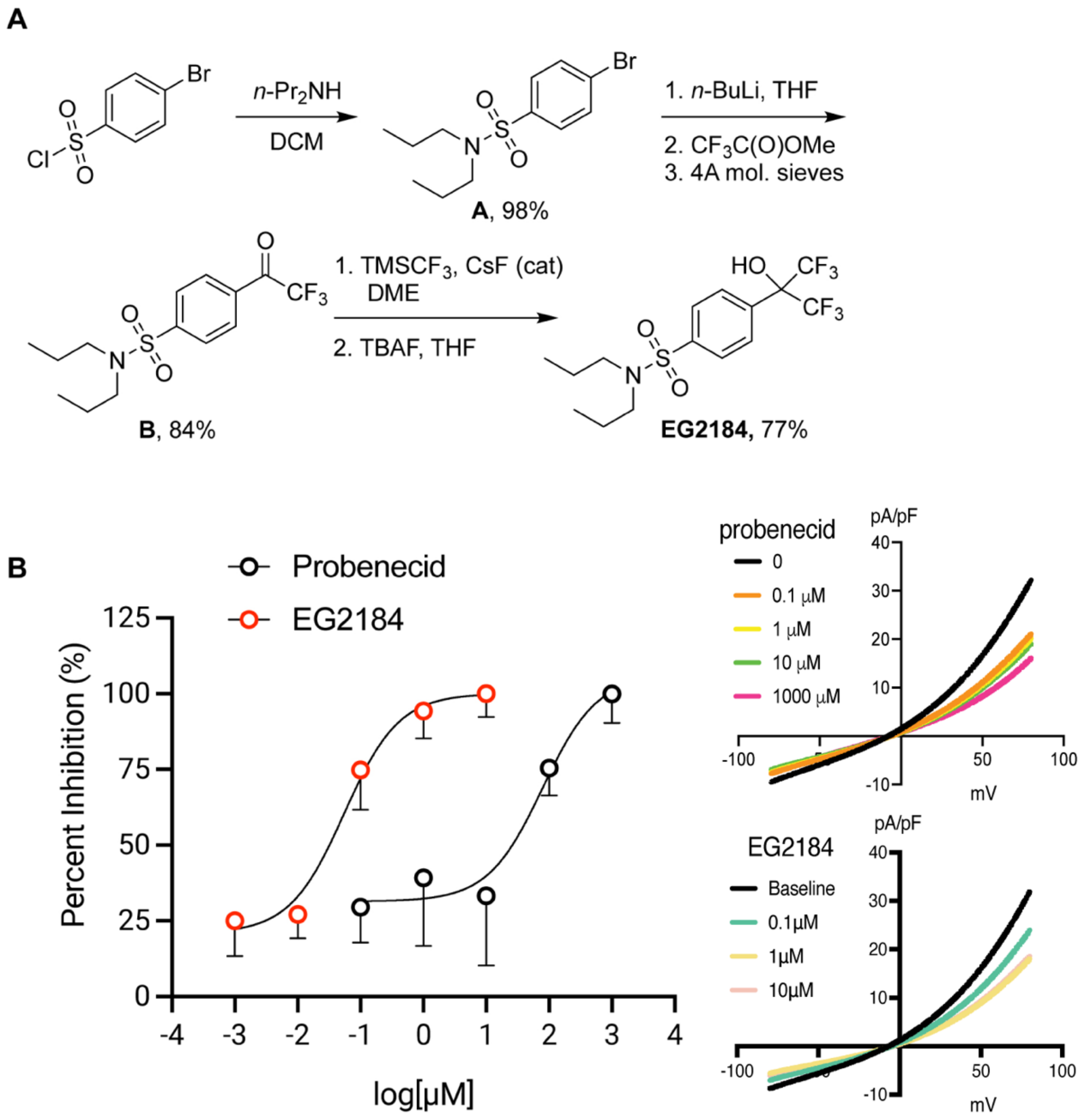

Supplementary Figure 15: Synthesis and Characterization of EG-2184.

(**A**) Synthetic scheme of EG-2184. (**B**) IC_{50} curves and traces of Probenecid (PRB) and EG-2184 (EG) compounds from whole-cell patch clamp recordings in Panx1 over-expressing HEK293T cells. Drug responses are plotted as percent inhibition of baseline Panx1 currents and normalized to highest concentration of drug used (100% inhibition). Concentrations range from 0.1 μM – 1000 μM for PRB (n=6, n=6, n=6, n=7, n=7 cells respectively), and 0.001 μM – 10 μM for EG (n=7, n=5, n=6, n=6, n=6 cells respectively). IC_{50} values for PRB (74.64 μM) and EG (0.054 μM) were calculated using a non-linear regression curve fit. All graphed data are presented as mean values ±SEM. Source data are provided as a Source Data file.

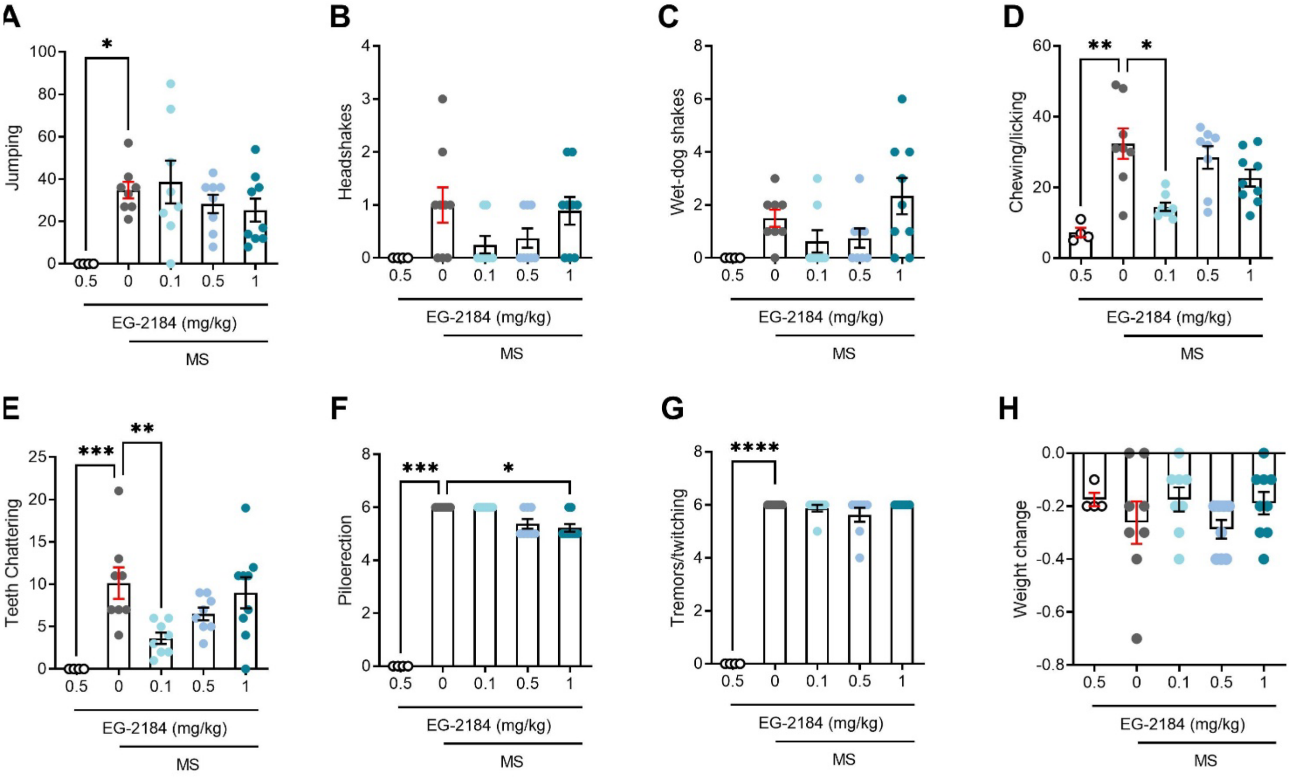

Supplementary Figure 16: Individual withdrawal behaviours in vehicle and EG-2184 treated mice.

Individual behaviour counts over a 30-minute period used to calculate cumulative withdrawal scores in vehicle (VEH) and EG-2184 (i.p.) treated morphine-dependent mice undergoing naloxone-precipitated withdrawal. (**A**) Total counts of jumping (one-way ANOVA, p=0.014). (**B**) Total counts of headshakes (Kruskall-Wallis test, p=0.063). (**C**) Total counts of wet-dog shakes (Kruskall-Wallis test, p=0.023). (**D**) Total counts of chewing and licking (Kruskall-Wallis test, p=0.0004). (**E**) Total counts of teeth chattering (one-way ANOVA, p=0.0006). (**F**) Presence of piloerection in each five-minute bin (Kruskall-Wallis test, p<0.0001). (**G**) Presence of tremors or twitching in each five-minute bin (Kruskall-Wallis test, p=0.0001). (**H**) Total weight change (Kruskall-Wallis test, p=0.3285). For all behaviours, VEH-saline N=7, VEH-morphine (MS), PRB-MS, 15 mg/kg, 25 mg/kg, 50 mg/kg: N=8 mice. All graphed data are presented as mean values ±SEM. *p<0.05, **p<0.01, ***p<0.001, ****p<0.0001. Source data are provided as a Source Data file.

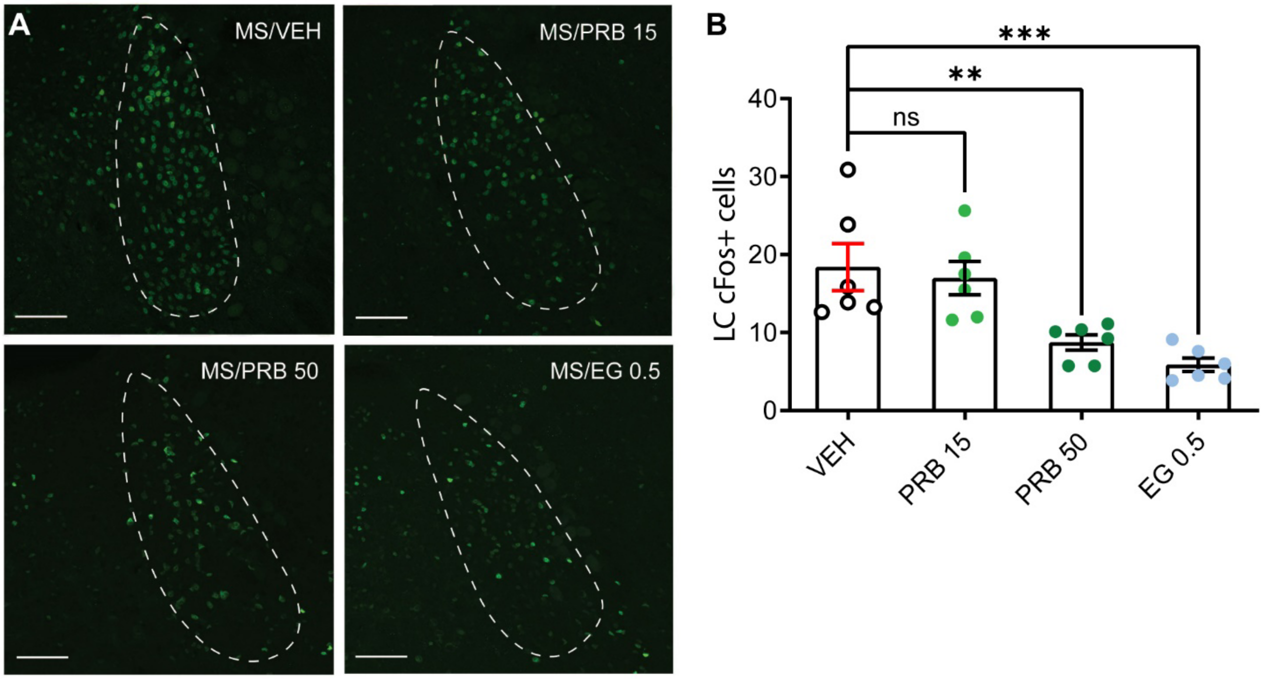

Supplementary Figure 17: Probenecid and EG-2184 reduce cFos positivity in the LC after naloxone precipitated opioid withdrawal.

(**A**) Representative images of cFos immunostaining in the LC during naloxone-precipitated withdrawal in morphine-treated (MS) mice treated with vehicle (VEH), Probenecid (PRB, 15 or 50 mg/kg, i.p.), or EG-2184 (EG, 0.5 mg/kg, i.p.). (**B**) Quantification of cFos+ cells in the LC (one-way ANOVA, p=0.0003, VEH, PRB 15, PRB 50, EG 0.5, N=6 mice each). All graphed data are presented as mean values ±SEM. *p<0.05, **p<0.01, ***p<0.001. Source data are provided as a Source Data file.

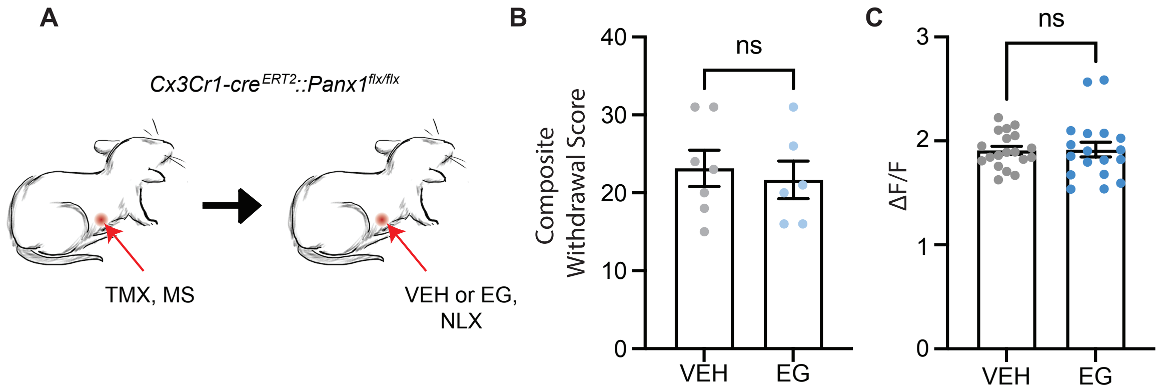

Supplementary Figure 18. Administration of EG-2184 does not further decrease withdrawal scores in microglial Panx1 deficient mice.

(**A**) Schematic of treatment paradigm. *Cx3cr1*-Cre^{ERT2}::*Panx1*$^{flx/flx}$ mice were first treated with tamoxifen (TMX), followed by a morphine withdrawal paradigm 28 days later. On the day of withdrawal, EG-2184 (EG, 0.5 mg/kg, i.p.) or vehicle (VEH) were administered 1 hour prior to naloxone precipitated withdrawal. (**B**) Effect of EG-2184 treatment in microglial Panx1 deficient mice (unpaired two-sided t-test, $p=0.6685$, VEH $N=7$, EG $N=6$ mice). (**C**) BzATP (100 μM)-evoked peak calcium response from BV2 microglia-like cells loaded with calcium indicator dye Fura-2AM treated with EG-2184 (10 nM) or vehicle (unpaired two-sided t-test, $p=0.922$, VEH $N=19$ cells from $n=3$ plates, EG $N=19$ cells from $n=3$ plates). All graphed data are presented as mean values ±SEM. Source data are provided as a Source Data file.

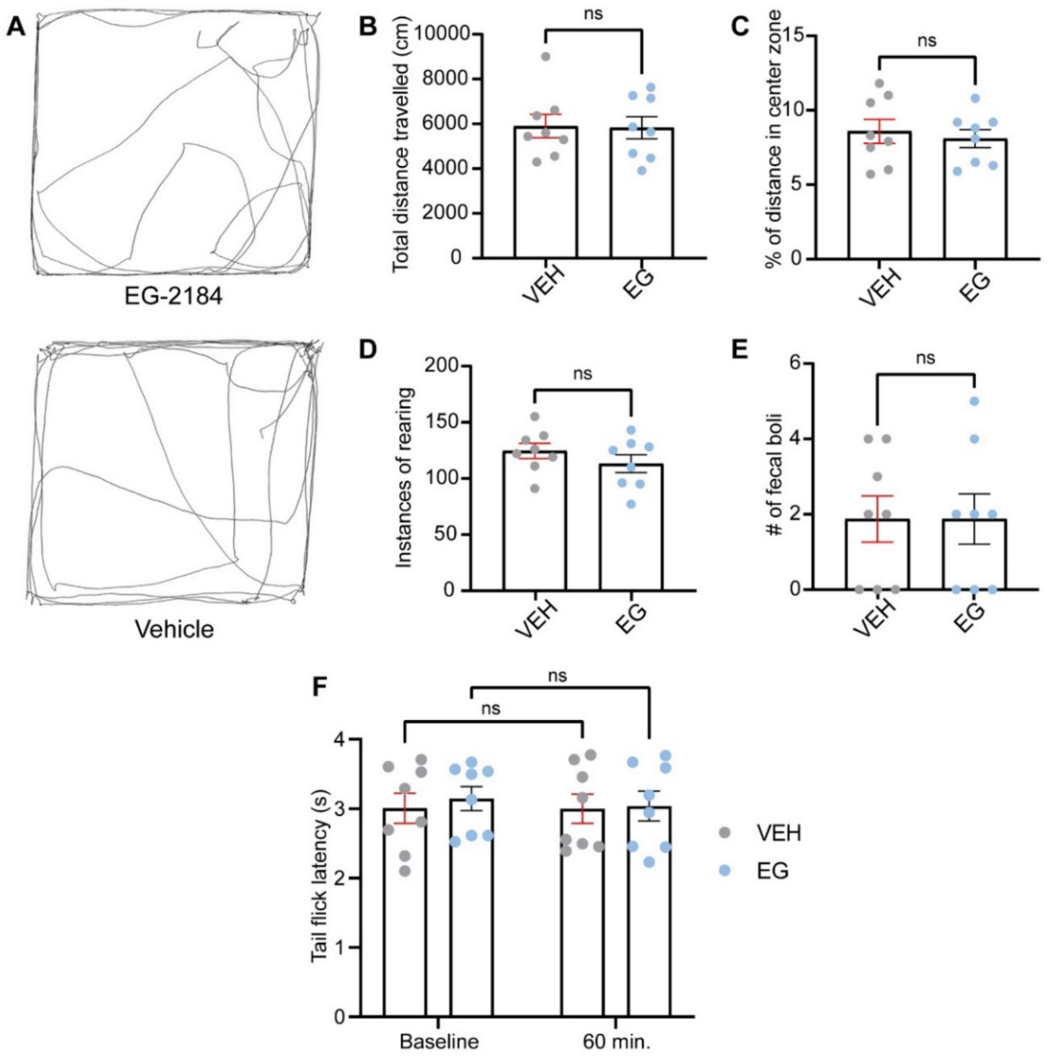

Supplementary Figure 19. Administration of EG-2184 does not alter locomotion, anxiety, or acute thermal nociception.

(**A**) Sample motion traces during 15-minute open field trials in C57Bl/6 mice injected with EG-2184 (EG, 0.5 mg/kg, i.p.) or vehicle (VEH). (**B**) Total distance travelled during open field trials (unpaired two-sided t-test, p=0.9233, N=8 mice in each group). (**C**) Percentage of distance travelled in the center zone (unpaired two-sided t-test, p=0.6376, N=8 in each group). (**D**) Instances of rearing (unpaired two-sided t-test, p=0.2925, N=8 mice in each group. (**E**) Number of fecal boli (unpaired two-sided t-test, p>0.9999, N=8 mice in each group. (**F**) Tail flick latency (two-way ANOVA, baseline vs. 60 minutes: p=0.4326; VEH vs EG: p=0.7585, N=8 mice in each group). All graphed data are presented as mean values ±SEM. Source data are provided as a Source Data file.

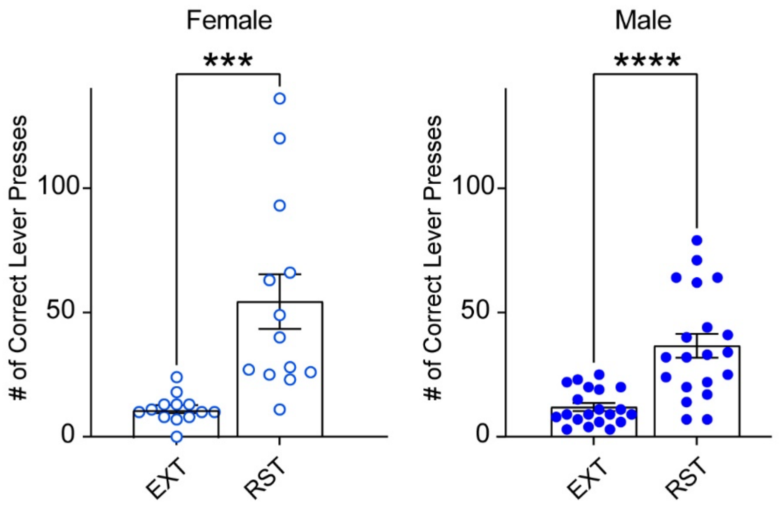

Supplementary Figure 20. Both male and female rats undergo reinstatement of morphine.

Separation of extinction and reinstatement trials into female (Wilcoxon matched-pairs signed rank two-sided test, $p=0.0002$, $N=13$ rats) and male cohorts (Wilcoxon matched-pairs signed rank two-sided test, $p<0.0001$, $N=20$ rats). All graphed data are presented as mean values ±SEM. ***$p<0.001$, ****$p<0.0001$. Source data are provided as a Source Data file.

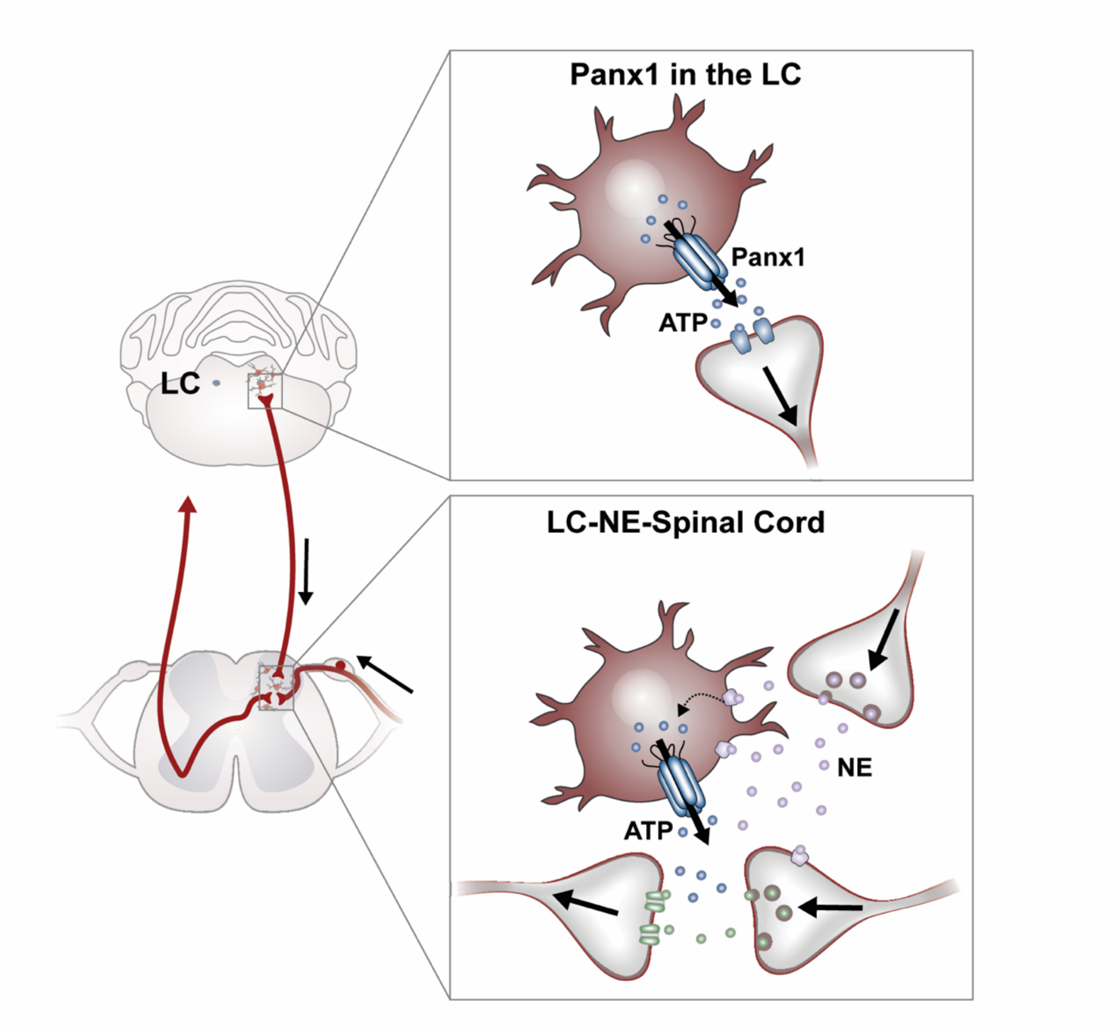

Supplementary Figure 21. Microglia Panx1 contributes to LC^{spinal} hyperexcitability during naloxone induced opioid withdrawal.

Microglia Panx1 channels release ATP in the LC and spinal cord to drive neuronal hyperexcitability during opioid withdrawal. Activity of spinally projecting LC neurons releases norepinephrine (NE). Genetic and pharmacological inhibition of Panx1, chemogenetic inhibition of LC^{spinal} neurons, or chemical depletion of LC noradrenergic neurons suppresses the neuronal hyperexcitation and alleviates opioid withdrawal. Treatment with probenecid or EG-2184 prevents the LC hyperexcitation, reducing opioid withdrawal, conditioned place aversion, and reinstatement.
