# Supplementary material for: Pannexin-1 channel inhibition alleviates opioid withdrawal in rodents by modulating locus coeruleus to spinal cord circuitry
Source: Nat Commun. 2024 Jul 24;15:6264. doi: 10.1038/s41467-024-50657-7 (PMC11269731; doi:10.1038/s41467-024-50657-7)
Supplement: Supplementary file 3 — Reporting Summary [file 41467_2024_50657_MOESM3_ESM.pdf]

nature portfolio

Corresponding author(s): *Double-anonymous peer review submissions: write DAPR and your manuscript number here instead of author names.*

Last updated by author(s): *YYYY-MM-DD*

Reporting Summary

Nature Portfolio wishes to improve the reproducibility of the work that we publish. This form provides structure for consistency and transparency in reporting. For further information on Nature Portfolio policies, see our Editorial Policies and the Editorial Policy Checklist.

Statistics

For all statistical analyses, confirm that the following items are present in the figure legend, table legend, main text, or Methods section.

n/a	Confirmed	
☐	☒	The exact sample size (*n*) for each experimental group/condition, given as a discrete number and unit of measurement
☐	☒	A statement on whether measurements were taken from distinct samples or whether the same sample was measured repeatedly
☐	☒	The statistical test(s) used AND whether they are one- or two-sided<br>*Only common tests should be described solely by name; describe more complex techniques in the Methods section.*
☐	☒	A description of all covariates tested
☐	☒	A description of any assumptions or corrections, such as tests of normality and adjustment for multiple comparisons
☐	☒	A full description of the statistical parameters including central tendency (e.g. means) or other basic estimates (e.g. regression coefficient) AND variation (e.g. standard deviation) or associated estimates of uncertainty (e.g. confidence intervals)
☐	☒	For null hypothesis testing, the test statistic (e.g. *F*, *t*, *r*) with confidence intervals, effect sizes, degrees of freedom and *P* value noted<br>*Give P values as exact values whenever suitable.*
☒	☐	For Bayesian analysis, information on the choice of priors and Markov chain Monte Carlo settings
☒	☐	For hierarchical and complex designs, identification of the appropriate level for tests and full reporting of outcomes
☒	☐	Estimates of effect sizes (e.g. Cohen's *d*, Pearson's *r*), indicating how they were calculated

Our web collection on statistics for biologists contains articles on many of the points above.

Software and code

Policy information about availability of computer code

Data collection	All data were collected and collated within Microsoft Excel or GraphPad Prism 9.4 or 10.2.0 (GraphPad Software, Inc., California)
Data analysis	All data were analyzed within GraphPad Prism 9.4 or 10.2.0 (GraphPad Software, Inc., California)

For manuscripts utilizing custom algorithms or software that are central to the research but not yet described in published literature, software must be made available to editors and reviewers. We strongly encourage code deposition in a community repository (e.g. GitHub). See the Nature Portfolio guidelines for submitting code & software for further information.

Data

Policy information about availability of data

All manuscripts must include a data availability statement. This statement should provide the following information, where applicable:

- Accession codes, unique identifiers, or web links for publicly available datasets
- A description of any restrictions on data availability
- For clinical datasets or third party data, please ensure that the statement adheres to our policy

Source data are provided with this paper.

Research involving human participants, their data, or biological material

Policy information about studies with human participants or human data. See also policy information about sex, gender (identity/presentation), and sexual orientation and race, ethnicity and racism.

Reporting on sex and gender	This study does not include any experiments performed on human subjects and therefore cannot encapsulate gender effects. Experiments were performed in both male and female mice. Where sex based differences were observed, they have been reported further within this document.
Reporting on race, ethnicity, or other socially relevant groupings	n/a
Population characteristics	n/a
Recruitment	n/a
Ethics oversight	n/a

Note that full information on the approval of the study protocol must also be provided in the manuscript.

Field-specific reporting

Please select the one below that is the best fit for your research. If you are not sure, read the appropriate sections before making your selection.

☒ Life sciences ☐ Behavioural & social sciences ☐ Ecological, evolutionary & environmental sciences

For a reference copy of the document with all sections, see nature.com/documents/nr-reporting-summary-flat.pdf

Life sciences study design

All studies must disclose on these points even when the disclosure is negative.

Sample size	Samples sizes for behavioural and immunohistochemical experiments were determined based on previous publications (Burma et al, 2017). Sample sizes for CPA and electrophysiological experiments were determined based on a pilot cohort to determine relative effect size, followed by power analysis to determine likely needed N for a significant effect.
Data exclusions	In CPA experiments, individual mice were excluded based on several possible criteria: traumatic naloxone or other drug injections on the day of behaviour, incorrect chamber placement in CPA experiments, absence of naloxone-precipitated withdrawal during conditioning in CPA experiments, or a preference for the naloxone-paired chamber in non-drug treated mice such that the CPA score exceeded +75. Two cohorts of mice were additionally excluded from CPA when positive littermate controls failed to show a CPA effect. Stress was identified as a likely factor due to fighting present in cages. In other behavioural experiments, mice were excluded if there were traumatic naloxone or other drug injections on the day of behaviour. In all DREADD experiments, mice were excluded if there was a lack of mCherry expression in the LC upon post-hoc analysis. In all electrophysiological experiments, cells were excluded from analysis if resting membrane potential exceeded -35 mV in neurons, or if access resistance was ≤20 or 25 MΩ (depending on neurons vs. HEK cells) at the start of the experiment or changed by more than 25% during the entire recording period. Slices were excluded from immunohistochemical or RNAscope analysis if Bregma could not be definitively identified, slices were torn, significant autofluorescence was noted as compared to other slices, or if air bubbles were present in mounted slides. In YO-PRO uptake assays, cells were excluded if they did not respond to an ionomycin challenge at the end of the experiment. All exclusions are explained further in depth within the methods section.
Replication	Key findings were replicated by multiple experimenters (e.g. CPA experiments were performed by two different experimenters, some immunohistochemical experiments were performed by up to three different experimenters). All experiments reported here successfully replicated, and results have therefore been pooled and presented as such.
Randomization	Mice treated with drug (morphine, probenecid, EG-2184, CNO, etc.) or control (saline, PBS, or vehicle) were run in parallel such that there was always at least one cage of drug treated mice and one cage of control treated mice within each experimental cohort. Littermates were used whenever possible, but cagemates could not be used as mixing for example morphine and saline treated mice within a cage can lead to fighting and injuries. In mice treated with probenecid, EG-2184, or CNO, experiments were performed with active drug and controls interspersed to control for time or litter effects. Choosing of which mice received drug or control was random. In CPA experiments, mice were assigned to drug or control groups to prevent biased preference prior to chamber pairing, and therefore could not be fully randomized.
Blinding	All electrophysiological experments were performed with the experimenter blinded either to drug condition or genotype of mouse, with unblinding occuring only after analysis. CPA experiments were performed with the experimenter blinded to expectation, but not to drug condition or genotype as pairing had to be even between groups (see randomization). Furthermore, CPA experiments were analyzed using an automated program (Ethovision) to reduce any potential bias. Immunohistochemical analyses were performed with the experimenter blinded to the drug condition or genotype. Behavioural experiments (withdrawal scoring and reinstatement) were all performed with the experimenter blinded to drug condition or genotype. Yo-pro assays were all performed with the experimenter blinded to drug condition.

Reporting for specific materials, systems and methods

We require information from authors about some types of materials, experimental systems and methods used in many studies. Here, indicate whether each material, system or method listed is relevant to your study. If you are not sure if a list item applies to your research, read the appropriate section before selecting a response.

Materials & experimental systems

n/a	Involved in the study
☐	☒ Antibodies
☐	☒ Eukaryotic cell lines
☒	☐ Palaeontology and archaeology
☐	☒ Animals and other organisms
☒	☐ Clinical data
☒	☐ Dual use research of concern
☒	☐ Plants

Methods

n/a	Involved in the study
☒	☐ ChIP-seq
☒	☐ Flow cytometry
☒	☐ MRI-based neuroimaging

Antibodies

Antibodies used

Primary antibodies: rabbit anti-cFos (ab190289, Abcam, lot#GR3379960-1), sheep-anti-TH (AB1542, Millipore, lot#3091702), rabbit anti-Iba1 (019-19741, Wako, lot#LEF4660), Mouse anti-NET (ab211463, Abcam, lot#GR3273270-2)

Secondary antibodies used: Donkey anti-rabbit Alexa 488, (A21206, Invitrogen, lot#2289872), Donkey anti-sheep Alexa 647, (A21448, Invitrogen, lot#2155286), Donkey anti-rabbit Alexa-647, (A31573, Invitrogen, lot#2420695), Donkey anti-mouse Alexa-568 (abcam, ab175472, lot#GR3213513-1)

Validation

ab190289 - Suitable for: IHC-FrFl, ICC, WB, IHC-P, Reacts with: Mouse, Rat, Human, Recombinant full length protein corresponding to Human c-Fos aa 1 to the C-terminus. expressed in and purified from E. coli., positive control in WB: HeLa and rat cortical membrane cells.

AB1542 - Suitable for: IHC, WB, Reacts with: Mouse, Rat, Mammals, Immunogen is Native tyrosine hydroxylase from rat pheochromocytoma. Positive control in WB: Routinely evaluated by Western Blot on mouse brain lysates (1:1000)

019-19741 - Suitable for: ICC, IHC(Frozen), Reacts with: Human, Mouse, Rat, antibody is raised against synthetic peptide corresponding to C-terminus of Iba1. product sheet states "We confirm this product has as good quality as that of the previous lot by immunostaining test"

ab211463 - Suitable for: IHC-P. Reacts with: Mouse, Rat, Human, immunogen is Recombinant fragment corresponding to Human Noradrenaline transporter aa 150-250. Positive control is IHC-P: Rat locus coeruleus and cerebellum, mouse cerebral cortex and hippocampal formation, human prostate, placenta and locus coeruleus tissues.

Eukaryotic cell lines

Policy information about cell lines and Sex and Gender in Research

Cell line source(s)

https://www.cedarlanelabs.com/Products/Detail/CRL-3216?lob=AllProducts - 293T; Embryonic Kidney Cells; Human (Homo sapiens) ATCC # CRL-3216

Authentication

None of the cell lines used were authenticated.

Mycoplasma contamination

Cell lines were not tested fro mycoplasma contamination.

Commonly misidentified lines
(See ICLAC register)

N/A

Animals and other research organisms

Policy information about studies involving animals; ARRIVE guidelines recommended for reporting animal research, and Sex and Gender in Research

Laboratory animals

Long Evans rats (male and female, 8-10 weeks of age) were bred in-house at Washington University. All C57Bl/6 mice (male and female, 7-14 weeks of age) were obtained from the Jackson Laboratory and housed at the University of Calgary. For some experiments, the following transgenic mouse lines were also obtained from the Jackson Laboratory and housed at the University of Calgary: B6.129P2(Cg)-Cx3cr1tm2.1(cre/ERT)Litt/WganJ (JAX #021160), GAD2-cre: Gad2tm2(cre)Zjh/J) (JAX #010802), and Ai9: B6.Cg-Gt(ROSA)26Sortm9(CAG-tdTomato)Hze/J) (JAX #007909). GAD2-cre mice were crossed with Ai9 mice to generate GAD2::Ai9 mice to express mCherry in GABAergic neurons.

Wild animals

N/A

Reporting on sex

As noted in a previous publication (Burma et al, 2017, Nature Medicine), no sex differences were observed in the role of Pannexin-1 in opioid withdrawal. We therefore designed our experiments to include both sexes where feasible (For example CPA could not be completed with both males and females in a single cohort as several mice are tested simultaneously). In experiments where both males and females were included, we separately tested and confirmed no evidence for a sex difference in effect in opioid withdrawal or reinstatement, and therefore displayed data with pooled sexes. If desired by reviewers, we can provide sex-specific information. Since the effect on reinstatement had not been shown with respect to sex previously, these data have been split by sex in supplemental figure 15. With respect to sex differences at baseline in mice treated with EG-2184 in supplemental figure 14, we did note that females had significantly higher distance traveled in open field as compared to males, as well as significantly lower baseline tail flick latency, as previously shown in literature. Given that there were no differences with respect to treatment with EG-2184 itself, these data were presented as pooled by sex. Sex separated data is available if requested.

Field-collected samples

N/A

Ethics oversight

All procedures were approved by the University of Calgary and Washington University Animal Care Committees, and are in accordance with the guidelines of the Canadian Council on Animal Care and the US National Institutes of Health Guide for the Care of Use of Laboratory Animals.

Note that full information on the approval of the study protocol must also be provided in the manuscript.

Plants

Seed stocks

N/A

Novel plant genotypes

N/A

Authentication

N/A
